# Maternal Health Coping Strategies of Migrant Women in Norway

**DOI:** 10.1155/2015/878040

**Published:** 2015-03-17

**Authors:** Berit Viken, Anne Lyberg, Elisabeth Severinsson

**Affiliations:** Centre for Women's, Family and Child Health, Faculty of Health Sciences, Buskerud and Vestfold University College, P.O. Box 235, 3603 Kongsberg, Norway

## Abstract

The aim of the study was to explore the maternal health coping strategies of migrant women in Norway. The ethnic and cultural background of the Norwegian population have become increasingly diverse. A challenge in practice is to adjust maternal health services to migrant women's specific needs. Previous studies have revealed that migrant women have difficulty achieving safe pregnancies and childbirths. Data were obtained by means of 17 semistructured interviews with women from South America, Europe, the Middle East, Asia, and Africa. Qualitative content analysis was employed. One overall theme is as follows: *keeping original traditions while at the same time being willing to integrate into Norwegian society*, and four themes emerged as follows: *balancing their sense of belongingness; seeking information and support from healthcare professionals; being open to new opportunities* and *focusing on feeling safe in the new country*. The results were interpreted in the light of Bronfenbrenner's ecological model. To provide quality care, healthcare professionals should focus on the development of migrant women's capabilities. Adaptation of maternal health services for culturally diverse migrant women also requires a culturally sensitive approach on the part of healthcare professionals.

## 1. Introduction

Health issues of migrant populations are an important public health concern [1]. The term migrant covers both emigrants and immigrants. In some of the literature including this paper, it is used synonymously with immigrant defined as a person with two foreign born parents. [2]. The healthcare needs of migrant women have been an area of concern both nationally and internationally, with particular focus on issues of sexual and maternal health including access to maternity care as well as maternal and neonatal outcomes [3–5]. Bollini et al. [6] state that although varying considerably, the reproductive health of immigrant women in Europe is fairly unsatisfactory. Research indicates that many migrant women find it difficult to access optimal maternity care and have worse maternal outcomes than native born women [7–9]. In Sweden and Norway the reproductive health of immigrant women is similar to that of the native born population, while it is inferior in UK and Italy, which may be due to the fact that receiving countries have different attitudes to immigrants and that the health and welfare of immigrant communities vary [6].

Balaam et al. [9] found that migrant women in maternity care in Europe were concerned with preserving their integrity in the new country. Many struggled to find meaning, which was related to inadequate communication, lack of connection, striving to cope, struggling to ensure a safe pregnancy and childbirth, and maintaining bodily integrity. Some felt insecure and not taken seriously during childbirth. Their traditional norms were often ignored and interpreters were not used. Organizational barriers to maternity care also existed, as they were not designed for migrants or adjusted to their needs. On the other hand, caring relationships with healthcare personnel could be a source of strength.

Although migrant women face many challenges, they also encounter opportunities in their new country of residence. Appropriate maternity care presupposes recognition of and feedback from immigrant women [10]. According to McCourt and Pearce [11], minority ethnic women share similar fundamental expectations of maternity care services to those of the wider population of which they are a part, but they experience a greater dissonance between expectations and experiences. Small et al. [12] found that immigrant women were less positive about maternity care than nonimmigrant women, although few differences were found in terms of their wishes. Migrant women focused on the need for respectful care, assistance with communication, and better information about how care is provided in their new country. According to Heaman et al. [13], migrant women are more likely to have inadequate prenatal care and the factors that contribute to their prenatal care utilization are poorly understood.

Research on migrant women in maternity care has traditionally focused on the reduction of risk during pregnancy and adverse birth outcomes [14], as well as the difficulties involved in caring for these women [15]. Although there has been little interest in the coping strategies of migrant women, some research has been carried out in this area. For example, Gagnon et al. [16] demonstrated that migrant women in Canada draw on a wide range of coping strategies and resources to respond to maternal-child health and psychosocial concerns. Among the 16 participants in the study, social inclusion was identified as an overarching factor for enhancing resiliency, while Margioula-Siarkou et al. [17] revealed that immigrant women had better obstetric and neonatal outcomes compared to native women in Greece.

The increasing diversity of the population in many countries presents a challenge for healthcare professionals [18]. Correa-Velez and Ryan [19] discussed the development of the best practice model of refugee maternity care comprising continuity of care, quality interpreter services, educational strategies, and psychosocial support. In Sweden, women from the Middle East and Sweden who were interviewed three months postpartum were found to focus on well-being and ability to care for the baby [20]. However, beliefs about health and illness differ and affect risk awareness as well as self-care practice. The researchers concluded that professionals play a significant role in supporting women and their families during the transition to motherhood, particularly those who are migrants. Identifying individual beliefs is of crucial importance [13]. A critical factor for women's experiences at all stages of care is the nature of the women's interactions with caregivers [20].

### 1.1. Background

Norway has experienced a strong growth in immigration in recent decades. The number of immigrants living in Norway has more than doubled since 2004 to 600,000 persons in 2013, corresponding to 12% of the population [22]. Immigrants bring a wealth of cultural, religious, and linguistic diversity [23]. Norwegian immigrants mainly come from Pakistan, Iraq, Vietnam, Somalia, Bosnia and Herzegovina, Iran, Turkey, Serbia, Sri Lanka, and Poland. As a result, Norwegian society has become more multicultural than ever before in the sense that different ethnic groups are a natural part of the same society. The growth in the number of immigrants and the fact that most immigrant women are of child-bearing age explain why an increasing number of babies born in Norway have an immigrant mother. In 2012, 23% of newborns, nearly one in four, had an immigrant mother [22].

Equal rights are enshrined in Norwegian legislation enabling immigrants and asylum seekers to access health care and medical treatment. The Patients' Rights Act [24] is intended to ensure that all citizens have equal access to good quality health care by granting patients' rights in their relations with the health service. The provisions of this Act are designed to promote a trusting relationship between the patient and the health service while safeguarding respect for life, integrity, and human dignity.

In Norway refugees and their relatives who joined them under the family reunification scheme have a right and duty to participate in the Introductory Programme [25], the purpose of which is to promote their participation in work and social life. Adult immigrants with a residence permit who are eligible for a settlement permit also have a right and duty to attend 300 hours of Norwegian language training and social knowledge. The provision of such training is the responsibility of the municipalities.

The authorities have issued guidelines for maternity care. The Norwegian National Clinical Guideline for Antenatal Care [26] states that women with a normal pregnancy should be cared for by a midwife or a general medical practitioner (GP). The woman herself should choose whether to be attended by a GP, a midwife, or a GP and midwife working in cooperation. The midwife in the community works in the maternal and child health clinic together with public health nurses. The National Professional Guideline for Postnatal Care [27] emphasizes equity in the postnatal health services, implying that healthcare personnel must respect diversity and recognize all individuals as equal members of society with the same right to health services. A study from Norway on healthcare professionals' perspectives revealed that equity in maternity care for migrant woman is related to managing and supporting educational, relational, and cultural diversity, about which public health nurses and midwives require more information and knowledge [18].

The maternal health coping strategies of migrant women should be viewed in the context of where they live. Psychologist Urie Bronfenbrenner [21] stated that human abilities and their realization depend to a significant degree on the larger social and institutional context. He presented an* ecological model*, which provides the framework from which researchers study an individual's relationship within communities and society in general. The model was originally used to explain how a child's development is influenced by different environments, but is now considered a valuable perspective in health promotion [28, 29]. In the present paper a description of the systems in the ecological model is provided in Section 2.5. In line with this theory, Thurston and Vissandjée [30] argue that the health of immigrant women is best understood in a framework that takes gender and the migratory experience into consideration, caring for the individual by focusing on social factors at meso- and macrolevels.

The salutogenic perspective means focusing on what creates health rather than taking a traditional medical approach that explores the roots of illness. The sociologist Aron Antonovsky posed the question: “What are the origins of health?” He explored the idea of health as a continuum from ease to dis-ease [31]. Migration as well as pregnancy and childbirth can be considered stressful life events. In a salutogenic approach, stressors are seen as challenges as opposed to something that damages life. Stressors are open-ended, initiating an interaction between the individual and her/his immediate environment. The emphasis is on the person's ability to use both internal and external General Resistance Resources (GRRs) to manage stressful situations. GRRs are found within people bound to their person and capacity, but also as material and nonmaterial resources in their immediate or distant environment. GRRs constitute the prerequisites for the development of a person's Sense of Coherence (SOC) [32]. SOC is a coping resource that enables a person to choose different strategies for solving different problems or managing life events. It is formed by the three dimensions comprehensibility, manageability, and meaningfulness [31]. GRRs and SOC can mutually reinforce one another.

The theory of salutogenesis corresponds closely with the key principles of the WHO Ottawa Charter for Health Promotion [33] and is useful in the making of healthy public policy [34]. The focus of this study was migrant women's movement on the health/dis-ease continuum and how to strengthen their ability to make the best choices for safeguarding health.

### 1.2. Aim

The aim is to explore the maternal health coping strategies of migrant women in Norway. The research questions were as follows: What are migrant women's expectations and experiences of the maternal and child health services? What are the maternal health coping strategies of migrant women?

## 2. Methods

### 2.1. Design

In this study a qualitative exploratory, descriptive design with a hermeneutic approach was employed [35, 36].

### 2.2. Participants

The study, in which 17 migrant women participated, was conducted at a maternal and child health centre by means of semistructured interviews. The participants had attended the centre during pregnancy and after childbirth during the previous year. All had given birth before the interviews took place. They were purposively selected and invited to take part in the study by a midwife at the health centre. The midwife contacted all women with a foreign background who had given birth during the previous year. The inclusion criteria were as follows: women who (1) were born in another country with two foreign born parents, (2) attended the maternity and child health centre, and (3) had given birth in the previous 12 months up to six weeks before the interview. There were no incentives to participate. The women represented the most common migrant populations in Norway. They comprised refugees, labour migrants, students, and women married to Norwegian men. Most of them spoke Norwegian, but Somali, Amharic, and Kurdish interpreters were employed in five of the interviews (Table 1).

### 2.3. Data Collection

The interviews were conducted between May and December 2012 at the participants' usual maternal and child health centre. Each interview lasted between 45 and 75 minutes, was audio-taped, and later transcribed verbatim. Most of the participants brought their baby and sometimes the father accompanied them. The interviewers (Berit Viken and Anne Lyberg) encouraged the participants to narrate their expectations and experiences of Norwegian maternity services and their life situation in general in the new country and as mothers in particular. Some examples of the questions posed are as follows: What are your experiences of maternity health care? Was the cooperation between you and the midwife, public health nurse, and doctor satisfactory? How did the health providers show respect for you? To what extent do you believe that your experiences and thoughts were taken seriously? Do you have any suggestions for changes in the health services? Who do you ask when you are uncertain about your own or your baby's health? What is it like living in this community? What do you do during the day? What makes you happy? The interviewers listened to the women's narratives and posed probing questions to further illuminate their experiences.

### 2.4. Ethical Considerations

The study was approved by the manager of the maternal and child health centre. The midwives and public health nurses working at the centre were also informed about the study, which was carried out according to the ethical guidelines for nursing research in the Nordic countries [37]. The Norwegian Social Science Data Services approved the study (number 30766). The participants were provided with written and verbal information about the purpose and design of the study by the midwife who tried to ascertain the women's ability to understand it. In addition, the written information was translated into English and Somali. A total of five women spoke Somali. The other women who needed a translation spoke different languages and received a verbal translation of the information from an interpreter. The women were advised about the voluntary nature of participation that their name and identity would not be disclosed as well as their right to withdraw at any time without any consequences in terms of maternal and child health care. They were also informed that the audio-tapes and transcripts would be destroyed at the end of the project. All data were stored in a locked and fireproof filing cabinet. As the researchers are responsible for the safety of the participants, the latter were invited to contact a named midwife at the maternity and child health centre if they in any way felt upset after the interview.

### 2.5. Analysis

A qualitative content analysis was conducted as described by Graneheim and Lundman [38]. It started with the authors reading the transcribed text to gain an overview of the data as a whole as well as an understanding of each interview. Thereafter, the two research questions “What are migrant women's expectations and experiences of the maternal and child health services?” and “What are the maternal health coping strategies of migrant women?” were focused on. Meaning units were identified and labelled with codes. The codes were compared, combined, reduced, and sorted into condensed meaning, subthemes, themes, and a main theme (Table 2).

To avoid misunderstanding the meaning of the text and ensure validity, the analysis moved back and forth between the emerging categories and the data. When interpreting the interview texts, the three authors discussed and shared the meanings arrived at, thus expanding and adjusting their individual understandings of the participants' expectations and experiences. This was continued until consensus was reached [39]. Thereafter the results of the analysis were interpreted in the light of Bronfenbrenner's ecological model [21]. Bronfenbrenner identified four environmental systems with which an individual interacts: the micro-, meso-, exo-, and macrosystem. The microsystem refers to the institutions and groups that most directly influence the child's development (or a person's adaptation, author's note), including family, school, religious institutions, neighbourhood, and peers. The mesosystem involves relations between microsystems and connections between settings. The exosystem comprises links between a social setting in which the individual does not play an active role and her/his immediate context. The macrosystem describes the culture in which individuals live. The national authorities are part of the macrosystem.

### 2.6. Methodological Limitations

The two interviewers are both public health nurses. This could have affected the participants' answers in that they might have wanted to be positive and not criticize the Norwegian health services. On the other hand, all participants had a residence permit in Norway and did not have a reason to fear that their answers could have negative consequences for them, as described by Liamputtong [40].

Another limitation was the language barrier. It became clear when the interviews started that some of the participants had not understood all the information provided beforehand. Informed consent presupposes that the individual understands the information and can make a voluntary decision to enrol and participate in the research. This can be a challenging task in cross-cultural research because “issues of culture come into play” [40]. The interviewers therefore used some time to explain further the purpose of the interview. When the participants afterwards were asked how it was to talk with the interviewer, they all had a positive attitude towards giving their opinions about subjects that were important to them.

## 3. Results and Interpretation

The interview data revealed the participants' strong will to integrate into the new society while at the same time maintaining traditions from their country of origin. The analysis revealed one main theme:* keeping original traditions and at the same time being willing to integrate into Norwegian society, *based on four themes,* balancing their sense of belongingness, seeking information and support from healthcare professionals, being open to new opportunities, *and* focusing on feeling safe in the new country. *The themes comprised several subthemes.

In the following the results will be presented and explained in the light of the four systems in the ecological model (Table 3).

### 3.1. Balancing Their Sense of Belongingness: The Microsystem

The first theme is seen in the light of the microsystem. The migrant women's life in a family and connections to other relatives and friends are part of the microsystem.

#### 3.1.1. Child-Centred Care

Several mothers stated that having a child gave them a feeling of belonging and that they followed the child's rhythm. They did not appear as occupied and multifocused as Norwegian new mothers. For example, one of the women could not understand why she should pump breast milk and keep it in a bottle because as she expressed*: “Why should I do that? The baby is always with me.”* (P3) Another woman just followed the child's rhythm.

*The day… passes so quickly, I breastfeed at least three times every night and sleep when the baby sleeps. I do not have much time to do other things than taking care of her. (P15)*

#### 3.1.2. Following Own Cultural and Religious Traditions as well as Norwegian Traditions

Faith in God was important for several of the migrant women and gave them confidence in daily life.

*I am comforted when I read the Koran, as you do not get any negative thoughts, just positive ones and trust. (P6)*

Other women stated that their belief in God is not “strong,” but that it is important to follow some religious traditions such as the celebration of Eid al-Fitr after Ramadan.

Several women had adopted Norwegian traditions such as going for walks. This is a very common activity among Norwegian families with children and everybody has a pram, but for migrants it can be a new experience.

*At weekends we normally take a walk in the forest. I love it. In my home country we cannot go for walks because it is too hot. You could not walk in the heat. (P1)*

Nearly all the women had changed their dietary habits and consumed “Norwegian” food, in that they ate more fish than in their native country. They also used a large amount of vegetables and fruit. Many took cod liver oil to prevent lack of vitamin D, which is a tradition in Norway and recommended for both mothers and children. At the same time many of the women said that they made use of advice from their country of origin, for example, herbal tea for the baby's stomach ache.

An Asian woman struggled with contradictory advice from healthcare professionals in Norway and from her mother and healthcare professionals in her native country. Her first pregnancy ended in miscarriage. She says that her GP had advised her to continue exercising as there was no need to change her lifestyle. She believed that exercise was the reason for her miscarriage because in her native country it is usual for women to be very careful during the first three months of pregnancy. Therefore it was confusing to receive different advice in Norway both from her Norwegian husband and the GP. When this woman became pregnant again, she travelled back to her country of origin and followed the advice not to exercise. She also brought some medicine back to Norway to prevent a miscarriage and this made her feel safer. (P14)

#### 3.1.3. Learning Norwegian, Establishing New Networks in Norway, and Maintaining Existing Networks Across Borders

The participants appreciated contact with other migrants but also longed for friendships with Norwegians. Some women found it difficult to get to know people in their new surroundings.

*I feel a bit lonely in this town. My midwife introduced me to a woman from Poland in the same situation as myself and she has become my friend now. But it is difficult to find Norwegian friends. It is important to be open and speak Norwegian. In another town, where I used to live, the health centre organised a group for women who had given birth, in which I felt very welcome. (P12)*

It was of major importance for all the migrant women to keep in contact with family and relatives in their country of origin, which they achieved by means of telephone and the Internet. They also travelled there on holidays or to a neighbouring country if they were unable to go back to their own country, especially in the winter, to experience sun and hot weather.* “Sunshine makes people healthy.” *(P11)

Many women highlighted family unity as the most important aspect of life.

*Unity in the family is the most important thing in life. Having gone through what we experienced, we never ever want to be separated again. (P5)*

A woman who had no family from her native country in Norway said that she received a great deal of help from her husband's family.

The women wanted to strengthen their sense of belonging to Norwegian society while at the same time maintaining their sense of belonging to their native country. One woman who came from a country during a war twenty years ago commented that she could not imagine living anywhere other than Norway now. However, her dream was to move back to her native country and be buried there.

### 3.2. Seeking Information and Support from Healthcare Professionals: The Mesosystem

The second theme represents the mesosystem. Migrant women's interactions with persons and institutions in the local community, such as the health services, make up the mesosystem.

#### 3.2.1. Trusting the Midwife

With the exception of one woman, all the participants had undergone several check-ups with the midwife during pregnancy and found the prenatal care very satisfactory. They experienced a positive caring relationship with the midwife. The information and support received helped them to cope with pregnancy and childbirth. Some women compared the health services in Norway with the poor health services in their native country. A woman from an African country plagued by war said:

* I think Norway is best when you are pregnant. The culture is very different to that of my home country and I miss my family, but I had very good discussions with the midwife, who made me relax and not think too much about the delivery. We have midwives in my country too, but we do not have a big health centre. Sometimes the mother or the child can die. But here in Norway it is the best you can get. The child check-ups, the hospital and the midwife are all very good. (P4)*

#### 3.2.2. Feeling Supported during Childbirth

The participants required knowledge about pregnancy to gain confidence and feel secure about their own and their baby's health. Many women had positive experiences of hospital staff and delivery ward routines. They were well informed and prepared for the birth, while many had visited the maternity ward before delivery. Most women felt listened to and well taken care of. Some said they were lucky to have had the same midwife at the pregnancy check-ups and at childbirth. This continuity of care created a feeling of security. The women who had experienced pregnancies or deliveries that required extra care were very grateful to be in Norway.

*I had long contractions and they helped me so that everything went well. They gave me love and care. I am thankful to the midwives who stood beside me the whole day and ensured the baby came out. They were so kind to me. If my child had been born in my home country he would not have survived. (P16)*

Some of the women were circumcised and needed surgery to open the vagina before delivery.

*It was my first child and I had just arrived in Norway. I needed a lot of information and help. I explained to the midwife that I wanted extra help during the delivery. She sent the information to the hospital and I got the help I needed to open the vagina before the baby came out. (P6)*

There were both good and bad experiences of childbirth in Norway, sometimes due to poor communication. One woman described the midwife as distant and absent. When this woman was in labour she described being treated with indifference and lack of respect, which she thought was because she was new in Norway and did not speak the language. “*When the baby was born they did not change my clothes or wash me. It was difficult for me.” *(P10)

#### 3.2.3. Following Guidance from Healthcare Professionals

The women considered it important to accept what was offered by the health services, also after childbirth.

*I am satisfied with the health centre. I got very good follow-up when I had difficulty with breast-feeding. When I had questions about the children, I received answers immediately. It is important that the children are checked by a doctor and vaccinated. I think the public health nurse follow-up is good. I do not miss anything. (P12)*

The women appreciated the information they received from the midwife during pregnancy and from the public health nurse in early motherhood. The information was provided during regular check-ups as well as open hours at the maternal and child health centre when there was no need to make an appointment. The women appreciated the opportunity to obtain advice when they needed it.

Some women need more follow-up from the maternal and child health centre because certain aspects of knowledge are less familiar to them compared with many ethnic Norwegian women. This can concern relatively minor issues such as different cultural traditions when the child is ready to eat solid food, for example, what sort of porridge they should start with. Language difficulties can accentuate problems with choosing foodstuffs in the shops. Another more serious matter is lack of knowledge about Norwegian legislation that prohibits hitting children. One of the participants had three children who were taken from her. She explained that she was new in Norway, did not speak the language, and did not know anything about Norwegian culture and parenting. She did not understand what to do and the child welfare service took her children into care. She has become very preoccupied with obtaining new knowledge and wants new immigrants to be informed about the right thing to do.

### 3.3. Being Open to New Opportunities: The Exosystem

The third theme is viewed within the framework of the exosystem. This system consists of decision makers (such as municipal officials and politicians) with whom the women have no direct contact but who still affect their lives. The participants adapted to Norwegian society in different ways. Many saw life in Norway as a new opportunity. Living in a welfare state allowed them an opportunity to learn Norwegian and study culture and society whilst receiving financial benefit. All the participants wanted to learn more Norwegian and obtain a higher educational qualification and at a later stage paid employment. They wanted their children to attend kindergarten and were impressed by the opportunities it presented to their children and themselves.

#### 3.3.1. Attending “The Introductory Programme”

Learning Norwegian as a part of the Introductory Programme was very important to all the participants. They wished to improve their language skills and have an opportunity to practise the language.

*I have a positive opinion of the municipality. I obtained all the information I needed about pregnancy and delivery. I also had the opportunity to learn Norwegian and earn money by attending “The Introductory Programme.” (P6)*

One woman appreciated not needing social security payment because her husband was employed and she was in receipt of introduction money.

#### 3.3.2. Starting New Activities

The migrant women wanted to work outside the home and were prepared to send their children to kindergarten. Nevertheless, not all of them could find suitable work. A woman from Africa had a job for several years in another European country, but she found it difficult to obtain a permanent position in Norway. She had been in trial placements but when they finished she did not obtain any further work.

*If you try you can manage anything, I just need a chance. If we are given a chance we will communicate and learn the language. I have many plans. I will go to high school next year and then my child will attend kindergarten. Then I will learn to drive and maybe start studying. (P8)*

### 3.4. Focusing on Feeling Safe in the New Country: The Macrosystem

The fourth theme is seen in the light of the macrosystem. The women emphasized their need to feel safe. Many came from countries in the throes of war and conflict with no public schools and social security.

#### 3.4.1. Comfort in a Safe Society and Social Security

When they longed for their family and native country, the thought of their own and their children's safe future in Norway helped them to manage their feelings.

*Sometimes when I think of moving back, I remind myself of how safe our future is in Norway. I think it is a good environment for children, with fresh air and clean, safe food. (P14)*

One woman who had lived for many years in Norway expressed her need to process memories from the war but also to move on.

#### 3.4.2. Being Aware of Differences between Norway and the Native Country

Culture influences one's view of health and disease. A woman from Asia explained how her opinion changed about the use of medications for the common cold.

*When I first came I was very irritated because I could not buy medication without a prescription. I thought why not? I did not understand. After a while I understood that we do not need medications if we just have a cold. Then I got used to it. Now I do not take medication very often. (P2)*

Other women wanted more examinations by specialists, for example, by a gynaecologist instead of a GP when pregnant and by a paediatrician to check their children in the maternal and child health centre. Several women wished for more ultrasound scans during pregnancy.

*I would have liked more ultrasound scans. Here I had only one. My best friend at home had ten ultrasound scans during her pregnancy. (P7)*

This woman paid for extra check-ups with a private doctor to obtain the ultrasound examinations she wanted and felt safer after those examinations.

## 4. Discussion

The aim of this study was to explore the maternal health coping strategies of migrant women in Norway. A qualitative content analysis was performed. The main theme that emerged was* keeping original traditions while at the same time being willing to integrate into Norwegian society.* The women seemed to manage these two apparently contradictory coping strategies. They had mostly positive experiences of the maternal health services, which were reflected in their willingness to integrate into society. In situations where they had negative experiences, the women used a wider range of strategies that involved keeping their original traditions and travelling to their native country to seek advice. The discussion will focus on migrant women's coping strategies in accordance with a salutogenic approach, including their expectations and experiences, both within and outside the maternal health services.

Sense of Coherence (SOC) appears to influence the women's actions and coping strategies when they become pregnant and after childbirth.* Balancing their sense of belongingness* to family members and institutions in Norway with that to their relatives in their country of origin affected the women's sense of meaningfulness, which is the motivational component of the SOC. The most meaningful task for the women is taking care of their own and the baby's health during pregnancy and childbirth. This is consistent with other studies which underline childbirth as a positive life event [41, 42].

Another important strategy is* being open to new opportunities, *which involves the willingness and efforts of migrant women to learn Norwegian by taking part in “The Introductory Programme.” Despite the fact that this programme is mandatory for all new refugees, the migrant women in our study were all very interested in it, especially learning Norwegian. The programme is provided in the community and is thus a part of migrant women's mesosystem. In the mesosystem all interaction takes place across settings [21]. Active participation in a new setting can increase the women's capabilities; for example language skills can help them to comprehend health information and social structures in Norwegian society. It also strengthens their social networks and makes their daily life more manageable by creating independency through not having to rely on translation by family members and interpreters. Ability to communicate in Norwegian also provides a stronger sense of belonging to society as it enables women to strengthen their social network and start new activities such as entering into paid employment. Nussbaum [43] contends that economic independence enhances women's self-respect and capacity for choice. Balancing their sense of belongingness can also be seen from a global perspective in accordance with Haith-Cooper and Bradshaw [44] and the model “The Pregnant Woman in the Global Context,” which emphasizes the environment of migrant women prior to, during, and after migration.

Another coping strategy is* seeking information and support from family and healthcare professionals*, where the women interact both within their microsystem and with the maternal and child health services, which are a part of their mesosystem. This strategy can be seen in connection with* personal agency*, the notion that the person has the capacity to make her (or his) own choices and exercise autonomy over personal life events and circumstances [45]. Information and support in pregnancy increase women's comprehensibility and manageability. Support during childbirth was also significant for their capacity building. However, some of the women felt exposed when meeting healthcare professionals as they experienced a lack of communication and social interaction. This has also been demonstrated in other researches [46].

The women also searched for information from the media both in Norway and other countries as well as from healthcare professionals and family in their native country. Similar findings are described in a study where Somali women said they received health information from, among other sources, an English television channel [47]. Most of the women in the present study maintained close links with family members in their country of origin through the internet, telephone, and/or international travel. This can be considered part of the macrosystem and is supported by other studies that have a transnational perspective on migration. Studies have revealed that migrants retain lasting ties with their country of origin. The identity and social practices of migrants transcend national borders [48, 49]. For example, a traditional medical perspective on pregnancy and childbirth was maintained by several of the participants, which contrasts with the concept of normal childbirth held by many Norwegian midwives and described in the international literature [50, 51].

The maternal health coping strategies of migrant women are also influenced by their* health literacy*, which is a composite term used to describe the capacities of persons to meet the complex demands related to health information in modern society [52]. It is a resource that equips people to navigate healthcare systems, critically assess information, and take greater control of their health. Two different concepts reflect health literacy, a clinical “risk” and a personal “asset.” Boerleider et al. [15] found that suboptimal health literacy was one of the difficulties midwives encountered when caring for nonwestern women in Netherlands, which can be said to represent the “risk” approach. In the present study we used the “asset” approach, in which health literacy applies to the development of skills and capacities that enable migrant women to exert greater control over their health and the factors that shape it. Consequently, health literacy affects women's maternal health coping strategies.

“The Introductory Programme,” maternal and child health services, and social media interact and contribute to the women's health literacy and thus their comprehensibility and manageability. Aasen [53] highlighted the importance of language skills for refugee women to cope with their daily life. Migrant women's comprehensibility also involves their original cultural traditions and formal education. Some of the participants in this study had poor education, which affected their health literacy. The women who could not read and write were largely dependent on verbal information from healthcare professionals to increase their comprehensibility. Consistent with these findings is the need for more innovative forms of communication that go beyond the written word [10, 47]. In their encounters with healthcare professionals the women who did not speak Norwegian were highly dependent on interpreters to achieve understanding. This is described as one of the main challenges in cross-cultural research [40].

An important coping strategy is* focusing on feeling safe in the new country*, which is connected to structures in Norwegian society and thus part of the macrosystem. When the women longed for their home country, they focused on the safety of themselves and their children. While this is related to Norway being a relatively peaceful country, it can also be seen as confidence in the welfare system. Experiencing comfort and social security in a safe society constituted a major part of the women's meaningfulness. Norway's welfare system, migrant, and refugee policy affect the social and economic conditions as well as the health services offered to migrant women. This is in accordance with the results of a group of researchers from Germany, Canada, and the United Kingdom [54], who compared migration and maternity care in the three countries. They found that while the design and delivery of maternal health services play a crucial role in ensuring positive experiences and good maternity outcomes for migrant women, these services must be understood within a much wider framework.

The main resources of migrant women in this study were their own capacity to readjust their lives, support from family and friends both in Norway and abroad, and information and support from healthcare professionals as well as financial support and educational opportunities provided by the municipality. Thus migrant women's maternal health coping strategies are affected by participation in the micro- and mesosystems as well as being influenced by the exo- and macrosystems.

## 5. Recommendations

Health promotion focuses on achieving equity in health. Reorienting health services is an important strategy in the Ottawa Charter for Health Promotion [33] and implies that the health sector should move in a health promotion direction, beyond its responsibility for providing clinical and curative services. It is vital that this leads to a change of attitude towards the organization of health services, where the focus is on the total needs of the individual as a whole person. The results of this study reveal that migrant women's maternal health coping strategies involve multisetting participation and can be understood by means of a system approach.

We advocate a salutogenic approach to maternity care research in order to strengthen the capabilities of migrant women to take care of their own and their baby's health. This will encourage healthcare professionals, policy makers, and maternity service users to consider maternity care from a health oriented and person centred perspective as opposed to one based on morbidity and mortality. Maternal healthcare services should therefore be adjusted to the needs of migrant women.

In order to provide culturally competent care, healthcare professionals must be culturally sensitive and aware of their own cultural background [55]. Cooperation with other sectors and multidisciplinary work can be part of an ecological approach to strengthen migrant women's capabilities and wellbeing.

## 6. Conclusions

This study had a salutogenic approach with focus on migrant women's resources and capabilities. It was concluded that migrant women were concerned with keeping their own traditions in pregnancy and childbirth while at the same time showing a willingness to integrate into Norwegian society. They used different coping strategies connected to family, healthcare professionals, and the education sector. In relation to ecological systems theory they thereby interacted with people in different environmental systems. They were also affected by the guidelines and decisions taken in the exo- and macrosystems. To provide quality care, healthcare professionals should use a system approach and focus on the development of migrant women's capabilities. Adaptation of maternal health services for culturally diverse migrant women also requires a culturally sensitive approach on the part of healthcare professionals.

## Figures and Tables

**Table 1:** Research participants, region of origin, age, number of years in Norway, education and work, number of children, and use of interpreter.

Research participant number	Region of origin	Age	Years in Norway	Number of children	Education and work	Interpreter
1	South America	35	8	2	Higher education, permanent job	
2	Europe	26	3	1	Student, higher education	
3	Europe	32	6	1	Professional education, permanent job	
4	Middle East	24	4	2	Secondary school	
5	Europe	33	26	2	High school, permanent job	
6	Africa	27	1	1	“Some years” of primary school	Yes
7	Europe	32	4	1	Higher education, permanent job	
8	Africa	38	5	8	Five years of primary school	Yes
9	Europe	37	18	4	High school, permanent job	
10	Africa	32	2.5	5	Two years of primary school	Yes
11	Africa		7	3	Six years of primary school	
12	Africa	37	8	2	Four years of primary school	Yes
13	Europe	28	20	1	Higher education, permanent job	
14	Asia	36	3	1	Higher education	
15	Africa	21	1.5	1	A “few years” of primary school	
16	The Middle East	20	3	1	Primary school	
17	The Middle East	25	1.5	1	No formal education	Yes

**Table 2:** Example of analysis: meaning unit, condensed meaning, subtheme, and theme.

Meaning unit	Condensed meaning	Subtheme	Theme
*I was very satisfied with my check-ups in the town where I used to live. I had no problems giving birth to my two children. It was the same midwife both times. I asked if I could get her and I did. I went to both the doctor and the midwife many times. I don't remember how many. *	Went for check-ups both to doctor and midwife. Satisfied with the services.	Following guidance from healthcare professionals	*Seeking information and support from healthcare professionals *

*I don't have any relatives in Norway, but I don't miss them, as I phone my mother and family. I have a lot of female friends in Norway, both from my home country and Norway. We talk a lot, we often chat on the telephone all day. If you are alone you get stressed. I like having contact with people. *	No relatives in Norway, but having contact by telephone. Many friends in Norway. Like to have contact with people.	Learning Norwegian, establishing new networks in Norway, and maintaining existing networks across borders	*Balancing their sense of belongingness *

**Table 3:** Empirical findings and interpretation in the light of ecological theory (Bronfenbrenner 1979 [21]).

Main theme: keeping original traditions while at the same time being willing to integrate into Norwegian society			
Microsystem	Mesosystem	Exosystem	Macrosystem
Theme: *Balancing their sense of belongingness* Subthemes: Child-centred care Following own cultural and religious traditions as well as Norwegian traditions Learning Norwegian, establishing new networks in Norway, and maintaining existing networks across borders	Theme: *Seeking information and support from healthcare professionals * Subthemes: Trusting the midwife Feeling supported during childbirth Following guidance from healthcare professionals	Theme: *Being open to new opportunities * Subthemes: Attending “The Introductory Programme” Starting new activities	Theme: *Focusing on feeling safe in the new country* Subthemes: Comfort in a safe society and social security Being aware of differences between Norway and the home country
